# Exploring the HeLa Dark Mitochondrial Proteome

**DOI:** 10.3389/fcell.2020.00137

**Published:** 2020-03-05

**Authors:** Federica Marini, Victor Corasolla Carregari, Viviana Greco, Maurizio Ronci, Federica Iavarone, Silvia Persichilli, Massimo Castagnola, Andrea Urbani, Luisa Pieroni

**Affiliations:** ^1^Institute of Biochemistry and Clinical Biochemistry, Università Cattolica del Sacro Cuore, Rome, Italy; ^2^Department of Laboratory Diagnostic and Infectious Diseases, Fondazione Policlinico Universitario Agostino Gemelli IRCCS, Rome, Italy; ^3^Department of Pharmacy, University G. D’Annunzio Chieti, Chieti, Italy; ^4^Proteomics and Metabonomics Unit, IRCCS-Fondazione Santa Lucia, Rome, Italy

**Keywords:** mitochondria, mass spectrometry, proteome, sub-proteome, dark proteome

## Abstract

In the framework of the Human Proteome Project initiative, we aim to improve mapping and characterization of mitochondrial proteome. In this work we implemented an experimental workflow, combining classical biochemical enrichments and mass spectrometry, to pursue a much deeper definition of mitochondrial proteome and possibly mine mitochondrial uncharacterized *dark proteins*. We fractionated in two compartments mitochondria enriched from HeLa cells in order to annotate 4230 proteins in both fraction by means of a multiple-enzyme digestion (trypsin, chymotrypsin and Glu-C) followed by mass spectrometry analysis using a combination of Data Dependent Acquisition (DDA) and Data Independent Acquisition (DIA). We detected 22 mitochondrial dark proteins not annotated for their function and we provide their relative abundance inside the mitochondrial organelle. Considering this work as a pilot study we expect that the same approach, in different biological system, could represent an advancement in the characterization of the human mitochondrial proteome providing uncharted ground to explore the mitonuclear phenotypic relationships. All spectra have been deposited to ProteomeXchange with PXD014201 and PXD014200 identifier.

## Introduction

In 2010, the international scientific community launched a joint effort for the comprehensive mapping of the Human Proteome. This program has been split in two sections named Chromosome-based Human Proteome Project (C-HPP), aimed at finding high-stringency evidence for all proteins encoded by the human genome, and Biology/Disease Human Proteome Project (B/D-HPP) whose mission was to annotate all the encoded proteins and to provide verified insights on their functions in health and disease (Paik et al., 2012).

The Italian Proteomics Association has dedicated its effort to extensively identify, characterize and quantify human mitochondrial proteome by proteomic mass spectrometry (MS) based technologies (Urbani et al., 2013).

The mitochondrial organelle is characterized by a peculiar structure composed of four sub-mitochondrial compartments (outer membrane, inter-membrane space, inner-membrane and matrix) through which mitochondrial protein carriers translocate to fulfill biochemical functions (Pfanner et al., 2019). Mitochondrion’s inner dynamics is a challenge for the achievement of a comprehensive mitochondrial proteome map suggesting the need to establish appropriate experimental strategies.

Several enrichment procedures are commonly used for the biochemical study of mitochondrial metabolic activity. In our previous work, we proposed how to assess the quality of a standard mitochondrial preparation in a proteomic workflow (Alberio et al., 2017).

Data on human proteins are integrated in the human–centric platform neXtProt, which includes all manually reviewed UniProtKB entries combined with related information derived from proteomics, transcriptomics and genomics data, to report intra- and inter-individual diversity (Gaudet et al., 2017). Proteins annotated in the neXtProt database, are categorized according to Protein Existence (PE) evidence levels. Based on experimental evidences, the PE level can be valued from 1 to 5, where PE1 stands for “experimental evidence at protein level,” while PE2, PE3, and PE4 imply some evidences regarding transcripts, protein homology or protein prediction studies but no proof of existence at protein level are present. Finally, PE5 value categorizes dubious genes, with poor prediction of protein production.

Although most of the human proteins have been annotated in the human proteome parts list, 10% of the complete human proteome lacks of functional information. The terms *dark proteins* and *dark proteome* have been recently adopted to describe proteins without an experimentally proven or homologically predicted functional annotation (Paik et al., 2018).

This study focused on improving the quality of mitochondrial proteome characterization, aiming to increase the recovery of integral membrane proteins.

We applied a combined biochemistry and MS based experimental protocol in order to investigate the mitochondrial proteome of HeLa cells.

Upon a two step membrane solubilization protocol, followed by an extensive MS analysis, we identified 3,187 and 3,275 proteins, in the two solubilized fraction F1 and F2, respectively. The qualitative evaluation of those IDs list, returned 4,230 proteins uniquely identified in the whole mitochondria enrichment.

According to the specific Integrated Mitochondrial Protein Index (IMPI Q2 2018) web resource (Smith and Robinson, 2019) 1,014 of them are formally annotated as mitochondrial proteins (mt-proteins). Among those mt-proteins, we extracted 22 uncharacterized PE1 (uPE1) dark proteins which have been relatively quantified in this work.

In this report we demonstrate that such an experimental workflow allows a deeper mapping of mitochondrial proteome providing a novel ground to depicting the mitonuclear genomic relationship. The present work draws an initial framework to search for uncharacterized mitochondrial proteins in different biological and pathological models that might precede the development of functional studies.

## Materials and Methods

This section is described in details in Supplementary Material.

## Results

### Experimental Workflow to Conduct Mitochondrial Proteome Analysis

We have implemented a combined biochemistry and MS based experimental workflow to extensively characterize the mt-proteome.

Briefly, after isolating the mitochondria from HeLa cells (Alberio et al., 2017), we performed a sub fractionation aiming to solubilize as much as possible the mitochondrial membrane, treating samples with two mild non-ionic detergents.

To begin with, we treated mitochondria with digitonin detergent, and we were able to separate a fraction of soluble proteins, that we named Fraction1 (F1), from a denser pellet, containing presumably more hydrophobic mt-inner membrane. We then treated this pellet with n-dodecil-β-(D)-maltoside to solubilize the remaining mt-membrane, and we called this protein extract Fraction 2 (F2). A bottom up approach was then used for MS analysis, which involves analysis of proteolytic peptides released upon enzymatic digestion. To further increase the proteome coverage, each sample was subject to three different proteolytic digestions and mass spectra have been acquired applying two different acquisition methods: Data Dependent Acquisition (DDA) and Data Independent Acquisition (DIA).

In particular, we used trypsin, chymotrypsin and Glu-C enzymes for both protein fractions F1 and F2. Digested peptides have been analyzed by LC-MS DDA on a Orbitrap Elite mass spectrometer (Thermo) and HDMS^E^, a DIA procedure, on a Synapt G2S*i* Q-TOF mass spectrometer with T Wave cell for ion mobility separation (Waters Corp.). DDA and DIA MS data have been processed independently using Peaks studio v7.5 and PLGS 3.0.3 software, respectively, and protein identification has been obtained by searching the human database neXtProt (2019-01-11).

Three technical replicates have been acquired for each of the twelve experimental condition (i.e. 2x sub-fraction/3x proteolytic digestion/2x acquisition protocol).

Combining the IDs list of proteins detected both in DIA and DDA experiments, we retrieved a list of 3,187 protein IDs from F1 and 3,275 total entries from F2.

These numbers have been obtained by merging, for each fraction, the list of proteins identified in DDA and DIA experiment performed in triplicates, on the three different proteolytic digestions, with the exclusion of redundant forms.

Finally, if we compare proteins identified in F1 and F2, after deduction of duplicates, we end up with a dataset of 4,230 protein IDs uniquely identified in whole mitochondria enrichment.

The list of all the proteins identified in DDA experiments are reported in Supplementary Tables 1–3, while Supplementary Tables 4–6 list all the protein IDs identified in DIA experiments. Supplementary Table 7, contains the list of the protein uniquely identified in F1, F2 and whole mitochondria.

**FIGURE 1:**
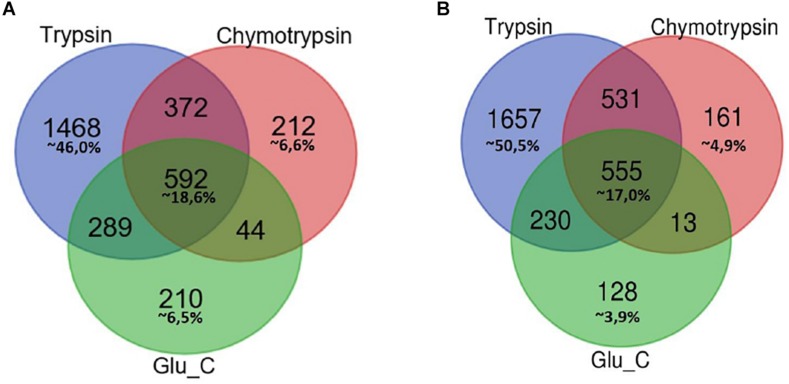
Venn Diagram of protein identification obtained by multiple enzyme digestion approach. Venn diagrams represent the overlap of the lists of proteins IDs detected by LC-MS/MS experiments upon proteolytic digestion with the three enzymes trypsin, chymotrypsin and GluC. The number of IDs uniquely identified by trypsin, Glu-C and chymotrypsin enzymatic digestion and/or number of proteins identified by two or three alternative and independent enzymatic digestion are indicated, respectively, in F1 **(A)** and F2 fractions **(B)**.

In Figure 1 Venn diagrams represent the distribution of protein identifications for each proteolytic digestion performed on F1 and F2 protein extracts.

In both cases the highest percentage of unique identifications arise from the analysis of tryptic peptides (46% of F1 in panel A, 50,5% of F2 in panel B), while a small percentage of proteins (less than 20%) has been retrieved in each of the three different batches of proteolytic peptides.

As might be expected, small groups of proteins identification are equally associated to peptides spectra obtained from two proteolytic digestions.

This multi-enzyme approach led to a considerable higher output of MS spectra, resulting in specific identifications which would have been lost using a traditional single digestion protocol.

The different MS acquisition approach also led to an extension of the data set obtained, though the contribution of the DDA data set, in our experimental configuration, results in an increase <10% in the number of identified proteins respect to DIA dataset, as shown in Supplementary Figure 1.

To extract from these lists a subset of proteins whose mitochondrial localization is proved from strong experimental evidences, we interrogated the Integrated Mitochondrial Protein Index (IMPI Q2 2018) gene database (Smith and Robinson, 2019). This database contains a reference set of mammalian genes encoding for proteins known or predicted to be mitochondrial from experimental data.

In order to give an overall number of total mt-proteins identified in this work, we merged the mt-protein list obtained in F1 and F2 according to IMPI classification and, by excluding the redundant forms, we were able to retrieve 1,014 mt-proteins (Supplementary Table 8).

### Mapping and Quantifying the Mitochondrial uPE1 Proteins

In order to search our datasets for the presence of mitochondrial proteins not yet annotated for their functions, belonging to the neXtProt PE1 category (dark proteins or uncharacterized PE1-uPE1), we submitted our mt-proteins ID list to the neXtProt dataset query NXQ_00022 which is a selection of those entry, only. Among those proteins, we retrieved 22 of the previously defined mitochondrial proteins, listed in Table 1 which can be defined as mitochondrial uPE1 dark proteins.

**TABLE 1:** List of mt-dark proteins identified in HeLa dataset.

**Accession**	**Description**	**Mito evidence IMPI**	**Mito localization IMPI**	**HPA cell localization**	**Enzyme**	**Exp. fraction**	**Gravy score**
NX_O60941-1	Dystrobrevin beta	Predicted	Unknown	Mitochondria (A)	Try; Glu-C	F1, F2	−0.55
NX_Q3SXM5-1	Inactive hydroxysteroid dehydrogenase-like protein 1	Known	OM	Intracellular, Membrane (P)	Chym; Try	F2	+0.15
NX_Q4VC31-1	Coiled-coil domain-containing protein 58	Known	IMS	Nucleoli, Mitochondria (A)	Try; Chym; Glu-C	F2	−0.60
NX_Q56VL3-1	OCIA domain-containing protein 2	Known	Unknown	Mitochondria (E)	Try	F2	−0.26
NX_Q8IYQ7-1	Threonine synthase-like 1	Known	Matrix	Nuclear bodies (A), Mitochondria (A), Cytosol (A)	Try	F2	−0.13
NX_Q8NFV4-1	Protein ABHD11	Known	Matrix	Mitochondria (S)	Try; Chym	F2	−0.09
NX_Q96EX1-1	Small integral membrane protein 12	Predicted	Unknown	Mitochondria (A)	Try	F2	−0.53
NX_Q96C01-1	Protein FAM136A	Known	IMS	Mitochondria (A)	Try; Glu-C	F2	−0.43
NX_Q96ER9-1	Coiled-coil domain-containing protein 51	Known	Matrix	Nucleosome (S), Mitochondria (S) Centrosome (A)	Try; Glu-C	F2	−0.38
NX_P56378-1	6.8 kDa mitochondrial proteolipid	Known	IM	Mitochondria (S),Nucleoli (S)	Try	F1,F2	−0.02
NX_Q9GZT6-1	Coiled-coil domain-containing protein 90B	Known	Matrix	Mitochondria (E)	Try; Glu-C	F2	−0.55
NX_A8MTT3-1	Protein CEBPZOS	Known	IMS	Nucleoplasm (A)	Try; Glu-C	F2	−0.27
NX_Q9H4I3-1	TraB domain-containing protein	Known	OM	Nucleus (A), Mitochondria (A)	Try	F2	−0.21
NX_Q9UFN0-1	Protein NipSnap homolog 3A	Known	Matrix	not available	Try; Glu-C	F2	−0.37
NX_Q6P1 × 6-1	UPF0598 protein C8orf82	Known	Matrix	Nucleus (A)	Try; Chym	F2	−0.23
NX_Q8N2U0-1	Transmembrane protein 256	Predicted	Unknown	Vesicles (A)	Try	F2	+0.46
NX_Q8WVI0-1	Small integral membrane protein 4	Predicted	Unknown	Nucleoplasm (A), Mitochondria (A)	Try	F2	−0.54
NX_Q8WW59-1	SPRY domain-containing protein 4	Known	Matrix	Nucleoplasm (A)	Try	F1,F2	−0.07
NX_Q96BQ5-1	Coiled-coil domain-containing protein 127	Known	OM/IMS	Nucleus (S), Nucleoli (S)	Try; Chym	F1,F2	−0.72
NX_Q96DB5-1	Regulator of microtubule dynamics protein 1	Known	OM/IMS	Centrosomes (S),Actin filaments (S)	Try	F1,F2	−0.37
NX_Q96KF7-1	Small integral membrane protein 8	Known	OM	Vesicles (A)	Try; Chym	F2	−0.55
NX_Q9NU23-1	LYR motif-containing protein 2	Known	matrix	Cytosol (A)	Try	F2	−0.70

*Accession, neXtprot accession number of uPE1 mt-dark protein; Description, extended protein name; MitoEvidenceIMPI. Mitochondrial protein classification; MitoLocalization IMPI, protein localization within mitochondria according to IMPI; HPA cell localization, main protein location Approved, Predicted, Enhanced or Supported in Human Protein Atlas project; Enzyme, protease used to generate the identified proteolytic peptides; Experimental Fraction, experimental sample in which the corresponding protein has been retrieved; Gravy Score, Grand Average of Hydropathy, measure the hydrophobicity of a specific protein.*

The table report IMPI evidence for mitochondrial localization (predicted or known to be mitochondrial) and location within mitochondria plus the Human Protein Atlas^1^ (Thul et al., 2017) evidence of cellular localization. Moreover we calculated the Gravy scores (Grand Average of Hydropathy) of each one of those 22 proteins, to get a measure of the hydrophobicity and the actual membrane-bound correlation.

Most of them (20 out of 22) have a Gravy score below 0 meaning that are more likely globular (hydrophilic protein), on the contrary, the 2 of them with a score value above 0 are more likely membranous (hydrophobic) (Kyte and Doolittle, 1982).

Interestingly these last two proteins (NX_Q3SXM5-1 with GS:+0.15; NX_Q8N2U0-1 with GS:+0.46) have been both identified in sample fraction F2.

Moreover, despite the fact that not all the proteins have a complete annotation, the theoretical localization inferred from IMPI confirms that we successfully dissolved both the outer membranes (OM) and the inner membrane (IM) of mitochondria.

For 5 of them a localization is still unknown but if we look at, for instance, proteins theoretically localized in the mitochondrial matrix, we could detected 6 out of 8 exclusively in the F2 fraction (e.g., NX_Q8IYQ7-1, Threonine Synthase Like-1) and 2 out of 8, in both F2 and F1 (e.g., NX_Q6P1 × 6-1, UPF0598 protein C8orf 82), suggesting that the double detergent treatment allows to disgregate the complex inner mitochondrial membrane structure.

To assess that the information retrieved with this experimental workflow allows to achieve a much deeper mapping of mitochondrial proteome, we investigated the presence of these uPE1 proteins, in other mitochondrial proteome dataset.

To this aim, this dark protein IDs list has been matched to the ProteomeXchange dataset PXD007053, previously deposited by our group (Alberio et al., 2017) that contains mitochondrial proteomes from 10 different cell lines including HeLa. Those datasets have been obtained by a DDA MS experiment of proteins extracted from intact mitochondria and digested by trypsin only. In Table 2 we reported the presence and distribution of each uPE1 in the dataset PXD007053.

**TABLE 2:** Distribution of uPE1 mt-dark proteins identified in PXD007053 repository database.

**Accession**	**Description**	**BJ**	**HeLa**	**H28**	**Hek293**	**HepG2**	**HUVEC**	**MDA-MB 231**	**Thp1**	**U2OS**	**SH-SY5Y**
NX_O60941-1	Dystrobrevin beta	–	–	–	–	–	–	–	–	–	–
NX_Q3SXM5-1	Inactive hydroxysteroid dehydrogenase-like protein 1	–	–	–	√	√	–	–	–	–	–
NX_Q4VC31-1	Coiled-coil domain-containing protein 58	–	–		√	√	–	√		–	–
NX_Q56VL3-1	OCIA domain-containing protein 2	–	√	√	–	–	√	√	√	√	
NX_Q8IYQ7-1	Threonine synthase-like 1	–	–	–	√	–	–	√	√	–	–
NX_Q8NFV4-1	Protein ABHD11	–	–	–	–	√	–	√	√	–	–
NX_Q96EX1-1	Small integral membrane protein 12	–	–	–	–	–	–	–	–	–	–
NX_Q96C01-1	Protein FAM136A	–	–	–	√	√	–	–	√	–	–
NX_Q96ER9-1	Coiled-coil domain-containing protein 51	–	–	–	√		–	–	–	–	–
NX_P56378-1	6.8 kDa mitochondrial proteolipid	–	–	–	–	–	–	–	–	–	–
NX_Q9GZT6-1	Coiled-coil domain-containing protein 90B	–	–	√	–	–	–	–	–	–	–
NX_A8MTT3-1	Protein CEBPZOS	–	–	–	–	–	–	–	–	–	–
NX_Q9H4I3-1	TraB domain-containing protein	–	–	√	–	–	√	–	√	–	–
NX_Q9UFN0-1	Protein NipSnap homolog 3A	–	–	√	–	–	–	√	√	√	–
NX_Q6P1 × 6-1	UPF0598 protein C8orf82	–	–	–	√	√	–	√	√	√	–
NX_Q8N2U0-1	Transmembrane protein 256	–	–	–	–	–	–	–	–	–	–
NX_Q8WVI0-1	Small integral membrane protein 4	–	–	–	–	–	–	–	–	–	–
NX_Q8WW59-1	SPRY domain-containing protein 4	–	–	–	√	√	–	–	–	–	–
NX_Q96BQ5-1	Coiled-coil domain-containing protein 127	–	–	√	–	–	–	–	–	–	–
NX_Q96DB5-1	Regulator of microtubule dynamics protein 1	–	–	√	√	–	–	√	√	–	–
NX_Q96KF7-1	Small integral membrane protein 8	–	–	–	–	–	–	–	–	–	–
NX_Q9NU23-1	LYR motif-containing protein 2	–	–	–	–	–	–	–	–	–	–

*Overlay of the 22 uPE1 mt-dark protein list (right column) with our previous repository dataset (PXD007053) obtained from 10 different cell lines. The checkmark highlights the match between each protein listed in the right column to the dataset obtained from mitochondria enriched from the 10 cell line, as indicated in the top raw of the table.*

Interestingly, the majority of uPE1 dark proteins found in HeLa dataset with the experimental workflow we are describing here, were not detected in the same cell line in the repository dataset PXD007053. In particular, comparing these two HeLa datasets only one uPE1 protein out of 22 was found in both. Such a difference is well expected given the higher dimensionality of the current HeLa dataset, which has been collected using a number of specific experimental features. Nevertheless, the results are slightly different if we compare the dark uPE1 proteins of our dataset with those obtained from other cell lines, where we can retrieve a variable number of them: 6 in H28, 8 in Hek293, 7 in HepG2, 2 in HUVEC, 7 in MDA MB231, 9 in THP1, 2 in U2OS, 1 in SHSY5Y. This evidence proves that a specific experimental workflow to sub-fractionate mitochondrial is mandatory in order to identify a higher number of dark proteins, mainly in cell line models, such as HeLa, U2OS or SHSY5Y, whose complete proteome has been well characterized in the last years (Thul et al., 2017; Paik et al., 2018). In fact, an enrichment of the intact mitochondrial fraction was not sufficient to identify these very low abundant dark proteins, as reported.

To confirm the low abundance of those proteins we exploited the quantitative analysis performed in our DIA experiments with the PLGS 3.0.3 software (Waters Co) (Silva et al., 2006; Vissers et al., 2007), taking in consideration data derived only from trypsin spectra, the unique condition which allowed the identification of all the 22 mt- uPE1 dark proteins. In these experiments, every single technical replicate of each sample was spiked with a fixed concentration of a digestion peptide standard, the Enolase 1 proteins from *Saccharomyces Cerevisiae* (UniProtKB/Swiss-Prot AC: P00924) (Waters) and the PLGS 3.0.3 software algorithm returned the relative concentration of each identified protein with respect to this standard (in fmol).

**FIGURE 2:**
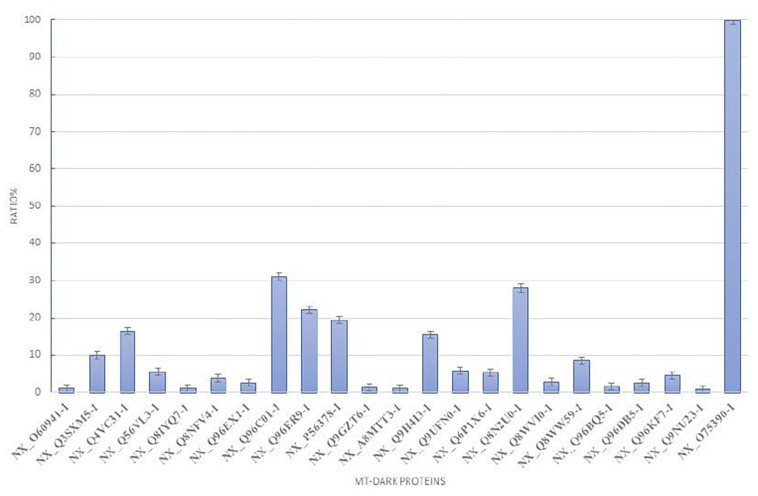
Relative abundance of mt-dark proteins in HeLa mitochondria enrichment. Relative label free quantification analysis of each mt-dark protein in comparison with Citrate Synthase (NX_075390-1)abundance expressed by percentage ratio and calculated from trypsin-DIA experiments.

To plot the abundance of each dark protein we normalized their concentration (in fmol) versus the concentration of Citrate Synthase (neXtProt Accession: NX_O7539-1), a typical mitochondrial protein marker (Eigentler et al., 2015), both calculated by the software respect to the spiked internal standard. In Figure 2, this quantification is plotted. To estimate the abundance of those uPE1 dark proteins in whole mitochondria, we calculated the average fmol amount of each mitochondrial dark proteins and Citrate Synthase, derived from three technical replicates of trypsin spectra acquired in DIA, in both fraction F1 and F2, then we plotted their ratio and relative STDV of the ratio. As expected, the histogram shows that the abundance of these uPE1 dark proteins is lower in comparison with a mitochondrial “gold standard” as Citrate Synthase. This result reinforces the idea that a specific sub-fractionation workflow could be a valuable tool to detect dark proteins inside the mitochondrial and cellular system.

## Discussion

Our previous work on mitochondrial proteomic standardization has established standard protocols for mitochondrial enrichment providing analytical key performance criteria (Alberio et al., 2017). Although our deposited dataset (PXD007053) represents an important contribution to the characterization of the mitochondrial proteome, only 300–600 mitochondrial proteins have been annotated in those dataset mostly belonging to the categories of the PE1 proteins in neXtProt PE evidence levels (Alberio et al., 2017) with only a small percentage of these IDs referring to mitochondrial membrane polypeptides.

With this work, using a higher dimensionality method, in term of proteome depth, we aim to overcome this limit and increase the information about mitochondrial proteome, possibly by increasing the detection of membrane proteins, and the likelihood of finding new dark proteins associated with mitochondria.

To this purpose, we conceived an experimental workflow combining detergent mediated membrane disruption with a deep proteomic investigation by means of LC-MS/MS label free bottom up experiments. For that matter, we pursued a multiple enzymatic protein digestion (i.e., trypsin, chymotrypsin and GluC) to generate peptides which have been subsequently analyzed both in DIA and DDA MS experiments.

We draw up all the experimental procedure in HeLa cells, though we are aware that they present some limitations (Liu et al., 2019). HeLa are the most common human immortalized cells used in research field with strong versatility and high durability. Their mitochondria pattern may reflect their lack of specific sub-cellular function in differential localization (Liu et al., 2019) and they provide a good example of isotropic mitochondrial proteome, resulting in a suitable cellular model to produce confident and high reproducible results (Masters, 2002; Liu et al., 2019). Lastly, they represent our reference standard for mitochondria enrichment procedure prior to mitochondrial proteomic analysis (Alberio et al., 2017), hence we retained that they represent an appropriate cellular model matching the aims of this work.

Firstly, we enriched mitochondria from HeLa cells. Then, we performed a sub-mitochondrial fractionation by means of incubation with specific detergents. We performed a first milder extraction with digitonin buffer and a subsequent solubilization of the most hydrophobic component by using n-dodecyl-β-(D)-maltoside. Both digitonin and n-dodecyl-β-(D)-maltoside are mild non-ionic detergents for the solubilization of biological membranes. In detail, digitonin is one of the mildest detergents used and for this reason we chose it to perform the first solubilization step. n-dodecyl-β-(D)-maltoside is more effective in solubilizing integral membrane proteins in native conformation and for this purpose we used it to solubilize and extract proteins from the lipophilic pellet (Wittig et al., 2006). Proteomic approach in sub-proteomes of cells and organelles increases the protein identification number and the reliability of the data, by reducing the complexity of protein and peptide mixture and consequently detecting less abundant species (Bradshaw and Burlingame, 2005; Bradshaw et al., 2005).

The multiple-enzyme approach provided a remarkable increase of the identification number. Indeed, cleavages at different amino acid residues (K, R for trypsin; F, W, Y for chymotrypsin; D, E for Glu-C), allow to maximize not only the protein coverage but also the number of IDs, as confirmed by the comparison with what was found previously in HeLa cells (Alberio et al., 2017). The combination of two different MS acquisition mode such as DIA and DDA leads also to a considerable increase of the overall entries.

The DDA mode provides selective, quantitative and sensitive analysis of peptides by defining transition list of the most intense precursor ions for further fragmentation. The main advantage of this method is represented by high accuracy and sensibility for select target but it leads to a lack of acquisition of proteins excluded from this transition list. To overcome this limit, we also operated in DIA mode which acquires spectra of all detectable precursors and their products by combining the sequential isolation of a large precursor window with full product ion spectrum acquisition (Messana et al., 2013; Vidova and Spacil, 2017). Moreover we could exploit the additional third dimension offered by the Ion Mobility Separation and perform Data Independent High Definition MS^E^ experiments (HDMS^E^) (Distler et al., 2014). DIA data provide us a more comprehensive information, sequencing all detectable peptides, including those not acquired in DDA mode.

Due to the different technologies and instrumental set up used in this work we do not predict a 100% overlap in the identification results. On the contrary we aim to enlarge the information. As a matter of fact we could confirm the identity of our entries by a high confident and significant overlap of 43.8.% between the DIA and DDA IDs for F1 fraction and for F2 of 45.3% as showed in Supplementary Figures 1A,B, with a contribution in terms of unique identification from DDA data below 10% and about 50% from DIA data.

Matching the total mitochondrial ID list obtained in this work (1,014 mt-proteins) with the current neXtProt database query for Dark Proteome (query: NXQ_00022), we were able to detect and quantify 22 mitochondrial uPE1 dark proteins As we reported in Table 1, the majority of these proteins are annotated with a localization in the IM and Matrix mitochondrial sub-compartments and they are expressed by genes allocated on different chromosomes. The experimental strategy adopted in this work represented the key tool to identify and detect these dark proteins in the mitochondrial organelle. In fact, as we showed in Table 2, we searched this selected 22 uPE1 list in another extensive mitochondrial dataset published from our group (Alberio et al., 2017) and most of them resulted to be absent. This aspect undoubtedly proves that sub-cellular and sub-mitochondrial proteome fractionation approach are mandatory in order to extensively detect uPE1 dark proteins at the mitochondrial level. We confirmed this suggestion by performing a comparative and quantitative analysis between the amount of each dark proteins and the Citrate Synthase protein. In Figure 2, the abundance of these dark proteins is lower in comparison with a very well expressed and typical mitochondrial protein standard as Citrate Synthase. Considering their small amount, their detection inside the spectra results to be very challenging due to MS signals suppressed by other more abundant proteins.

In conclusion this work represents a valid experimental MS based workflow able to extensively characterize the mitochondrial proteome by offering confident and comprehensive mitochondrial proteomic dataset repository publicly available which could be sources for further mitochondrial investigation studies on different experimental model.

Moreover, this work provides important and new insights about the mt-dark proteome. In this perspective proteomic studies may provide significant contributions to pharmaceutical research and may support the development of personalized medicinal applications.

## Data Availability Statement

The mass spectrometry datasets generated for this study can be found in the ProteomeXchange Consortium via the PRIDE (Perez-Riverol et al., 2019) partner repository with the dataset identifier PXD014200 and PXD014201.

## Author Contributions

FM, LP, and AU designed the experiments and wrote the manuscript. FM performed the cellular biology, biochemistry, and bio-informatic analysis. FM, VC, VG, MR, FI, and SP carried out the LC-MS experiments and data analysis. LP, AU, and MC revised the manuscript. All authors read and approved the final version of the manuscript.

## Conflict of Interest

The authors declare that the research was conducted in the absence of any commercial or financial relationships that could be construed as a potential conflict of interest.

## Footnotes

1 www.proteinatlas.org, v18.1
