# Supplementary material for: Exploring the HeLa Dark Mitochondrial Proteome
Source: Front Cell Dev Biol. 2020 Mar 5;8:137. doi: 10.3389/fcell.2020.00137 (PMC7066081; doi:10.3389/fcell.2020.00137)
Supplement: Supplementary file 1 [file Data_Sheet_1.docx]

Supplementary Material

# Supplementary Data

**Materials and Methods**

**Chemicals and Materials**

All cell culture reagents and materials such as: DMEM, Fetal Bovine Serum (FBS), Penicillin-Streptomycin (Pen/Strept), L-Glutammine, PBS, tissue culture dishes, plus Sucrose, (4-(2-hydroxyethyl)-1-piperazineethanesulfonic acid) (Hepes), ethylenediaminetetraacetic acid (EDTA), Bovine Serum Albumine (BSA), (D)-Mannitol, digitonin and n-dodecyl-β-(D)-maltoside used for mitochondria enrichment and fractionation, were purchased by Sigma Aldrich, Inc. (St.Louis, MO). Microcon-10 Centrifugal Filter are from Merck Millipore Ltd. (Cork, IRL) and urea, tris, dithiothreitol (DTT), iodioacetamide (IAA), ammonium bicarbonate (AMBIC) and formic acid (FA) all purchased from Sigma Aldrich (St.Louis, MO.). Modified trypsin, chymotrypsin and Glu-C enzymes were from Promega (Madison, WI) and water, acetonitrile (ACN), formic acid (FA) (OPTIMALC/MS Grade) for LC-MS analysis were purchased from Fisher Scientific, U.K.

**Cell Culture**

HeLa cells were maintained at 37°C, and 5% CO_2_ in a humidified incubator and grown in high glucose DMEM, supplemented with 10% FBS, 1% Penicillin-Streptomycin, 1% (L)-glutamine. Mycoplasma tests were routinely performed by EZ-PCR Mycoplasma Test Kit (Biological Industries) in order to check the absence of contamination.

**Mitochondrial Enrichment and Sub-fractionation**

Four mitochondrial samples, each enriched by 10^9 HeLa cells, have been prepared. HeLa cells have been harvested, washed and centrifuged at 600xg (10min, RT) in PBS buffer to remove all medium and trypsin residues. Cell pellet has been resuspended in Mitochondrial Buffer (MB: 0.21M (D)-mannitol, 0.07M sucrose, 1mM EDTA, 0.01M Hepes at pH 7.5, plus 1X Protease Inhibitor Cocktail, P8340 Sigma Aldrich -St.Louis, MO) and mechanically lysed by 30 strokes in a Teflon Glass Homogenizer.

The homogenate has been centrifuged at 600xg (10min, 4°C) to remove cell debris, nuclei and intact cells. Supernatant was centrifuged at 7.000xg (10min, 4°C) to collect crude mitochondria, which have been subsequently purified by ultracentrifugation at 96000 x g, 2hrs, 4°C on a discontinuous sucrose gradient in order to recover mitochondria fraction in the gradient interface, as previously described and in compliance with a standardized procedure ([Frezza et al., 2007](#_ENREF_4);[Alberio et al., 2017](#_ENREF_1)). In order to recover a fair amount of sub-mitochondrial fractions for the MS analysis, the mitochondrial enrichments were pooled together and stored at -80°C until use. To disrupt inner and outer mitochondrial membrane, we applied the protocol described by Nishimura, et al ([Nishimura, 2014](#_ENREF_10)), with slight modification. Briefly, the mitochondria pool has been resuspended in Digitonin Buffer (DB) freshly prepared and composed by MB added with 1mg/mL digitonin. The suspension has been mixed for 15 min by using a micro tube mixer at max speed and subsequently centrifuged at 10.000 x g (10min, 4°C). We recover the supernatant, or Fraction 1(F1) from the pellet (F2). The F2 has been resuspended in MB added with 1% of n-dodecyl-β-(D)-maltoside and ice cold sonicated in order to disrupt and solubilize also hydrophobic membrane. Finally, the suspension was centrifuged at 100.000 x g (30min, 4°C) and the supernatant containing F2 proteins has been collected.

**Mitochondrial Protein Sample Digestion**

The protein content of F1 and F2 fractions was quantified by Bradford assay (Bio-Rad). To perform enzymatic proteolysis three aliquots of 20 μg protein amount respectively of F1 and F2 fraction were transferred on Microcon-10 Centrifugal Filter, with 10kDa cut off, and processed following a FASP protein digestion procedure ([Distler et al., 2016](#_ENREF_3)). Upon filter aided buffer exchange to Urea Buffer (UB: 8M Urea, 100mM Tris-HCl in water, pH 8.5) denatured proteins were reduced by 8mM DTT in UB (15 min at 56°C) and alkylated by 0.05M IAA in UB ( 20min at RT).

We ended up with three aliquots of denatured protein ready for digestion for each mitochondrial sub fraction, F1 and F2 and, for each of them, we performed, in parallel, a proteolytic digestion with trypsin, chymotrypsin and GluC, at an enzyme:protein ratio of 1:50 (w/w), upon filter aided exchange to the appropriate digestion buffer solution.

In detail, trypsin and Glu-C digestions were carried out at 37°C, 16-18 hours in 0.05M AMBIC solution , whilst chymotrypsin digestion was led at 25°C, 16-18 hours in 0.1M Tris-HCl and 10mM CaCl_2_ pH 8.0. All digestions were blocked by adding FA to a final concentration of 0.2% (v/v) and the peptides were recovered from the filter in 0.05M AMBIC, concentrated in a speedvac and stored at -80°C until use.

**Liquid Chromatography - Mass Spectrometry**

Sub-mitochondrial fractions (F1 and F2) protein extracts digested by trypsin, chymotrypsin and Glu-C proteases have been analysed by LC-MS/MS.

All the spectra have been acquired in three technical replicates both in DIA and DDA experiments

***DIA experiments***

Mitochondrial peptides from each sample (F1 and F2) have been resuspended in 0.1% FA at a concentration of 1µg/uL and stored at -80°C until used.

Each injected technical replicate consists of 0.30 µg (0.06µg/µL) of digested peptides spiked with 500 fmol (100fmol/μL) of MassPREP Enolase Digestion Standard (Waters Corp.). The separation of tryptic peptides was then performed on an ACQUITY MClass System (Waters Corp.) by loading 5 µL of each digested samples onto a Symmetry C18 5 μm, 180 μm × 20 mm precolumn (Waters Corp.) subsequently separated by a 120 min reversed phase gradient at 300 nL/min (linear gradient, 2–40% ACN over 90 min) using a HSS T3 C18 1.8 μm, 75 μm × 150 mm nanoscale LC column (Waters Corp.) maintained at 40 °C. Gradient was obtained by using as mobile phases the following solution: A = 0.1% formic acid (FA) in water B = 0.1% formic acid (FA) in acetonitrile (ACN). Separated peptides have been analysed by High Definition Synapt G2-S*i* Mass spectrometer directly coupled to the chromatographic system. MS signals have been detected in High Definition MS^E^ (HDMS^E^) a data-independent acquisition (DIA) protocol where ion mobility separation (IMS) has been integrated into LC-MS^E^ workflow (Distler et al., 2016). The following mass spectrometer parameters have been used: positive survey polarity of electrospray source (ES+), acquisition mode mass range 50-2000m/z, capillary source voltage 3.2 kV, source T 80°C , cone voltage 40eV, TOF resolution power 20000, precursor ion charge state 0.2-4, trap collision energy 4eV, transfer collision energy 2eV precursor MS scan time 0.5 sec and fragment MS/MS scan time 1.0 sec. All spectra have been acquired in Ion Mobility Separation mode (IMS) cycles with wave height at 40 V, wave velocity of 650 m/s, transfer wave height 4V and transfer wave velocity of 175 m/s([Distler et al., 2014](#_ENREF_2);[Greco et al., 2018](#_ENREF_6)). Data were post-acquisition lock mass corrected using the doubly charged monoisotopic ion of [Glu1]-Fibrinopeptide B (Waters), sampled every 30 s.

***DDA experiments***

Mitochondrial peptides were suspended in 0.1% FA and the separation of peptides was performed on an Orbitrap Elite mass spectrometer (Thermo Scientific, San Jose,CA). 0.30μg of each sample was loaded onto a precolumn Acclaim PepMap100 C18, 5 μm, 100 Å, 300 μm i.d. x 5 mm (Thermo Scientific, San Jose, CA) and after 5 minutes of trapping was separated by a column Easy-Spray PepMap C18 (2 µm 100 Å 15 cm x 50 µm ID) with a Thermo Scientific Dionex UltiMate 3000 RSLC nano system (Sunnyvale, CA). For the gradient elution water-FA (99.9:0.1, v/v) has been used as eluent A and ACN-water-FA (80/19.9/0.1, v/v) as eluent B with the following chromatographic steps: (i) 5% of eluent B (7 min), (ii) from 5 to 35% of eluent B (113 min), (iii) from 35 to 99% of B (15 min), (iv) 99% of B (10 min), (v) from 99 to 5% of B (2 min), (vi) 5% of B for column conditioning (13 min). The procedure was carried out at 35°C column temperature with a flow rate 300nL/min and the injection volume was set at 5.0 μL. MS spectra were collected in positive Full Scan acquisition mode in the 350-2,000 m/z range and with a resolution power of 60,000. The nanoESI tuning parameters were: capillary temperature 250 °C, source voltage 1.5 kV, sheath gas 0, auxiliary gas 0, S lens RR level 50%. MS/MS analyses were performed in data-dependent scan (DDS) mode, a data dependent acquisition (DDA) protocol, by selecting and fragmenting the twenty most intense multiple-charged ions of the collected Full Scan spectra by using collision induced dissociation (CID, 35% normalized collision energy) with a resolution power of 60,000. Only precursors with a charge state 2-7 and an intensity above the threshold of 5x10^3^ were collected for MS/MS. The DDS set parameters were the following: repeat count 1, repeat duration 30 s, exclusion list size 500, exclusion duration 15 s, exclusion mass width relative reference mass in the low and high range 10 ppm, minimum signal threshold (counts) 500, default charge state 2, isolation width 2 (m/z), activation q 0.250, activation time 10 ms([Martelli et al., 2018](#_ENREF_9))

**MS Data Analysis**

In this research, the last updated release of neXtProt (2019-01) with a total of 19,823 neXtProt PE1,2,3,4 predicted human protein entries, excluding uncertain (PE5) genes([Lane et al., 2013](#_ENREF_8)) has been used as a reference database

Mass Spectrometry Data Interpretation was achieved in agreement with the HPP Mass Guidelines Version 2.1.0

***DIA data analysis***

Continuum LC-MS data from three replicate runs for each sample have been processed for qualitative and quantitative analysis using the software ProteinLynx Global Server v. 3.0.3 (PLGS, Waters Corp.)([Silva et al., 2006](#_ENREF_13);[Vissers et al., 2007](#_ENREF_16)).

The qualitative identification of proteins has been obtained by searching in Homo Sapiens database (neXtProt Protein reviewed release 2019-01)([Gaudet et al., 2017](#_ENREF_5)) to which the sequence of Enolase 1 proteins from Saccharomyces Cerevisiae (UniProtKB/Swiss-Prot AC: P00924) was appended. Search parameters were set as: trypsin, chymotrypsin and Glu-C specified enzyme for each sample according to the digestion performed, automatic tolerance for precursor ions and for product ions, minimum 1 fragment ions matched per peptide, minimum 3 fragment ions matched per protein, minimum 1 peptide matched per protein, 2 missed cleavage, carbamydomethylation of cysteines as fixed modification and oxidation of methionines as variable modifications. False discovery rate (FDR) of the identification algorithm was thresholded as ≤ 1% at protein level, based on a target decoy database. For quantitative analysis, every single technical replicate of each sample has been spiked by a fixed concentration (100fmol/µL) of a digestion peptide standard, the Enolase 1 proteins from Saccharomyces Cerevisiae (UniProtKB/Swiss-Prot AC: P00924)(Waters) and the PLGS 3.0.3 software algorithm return the relative concentration of each identified protein with respect to this standard ( in fmol and ng ).

***DDA data analysis***

Thermo raw data were processed using PEAKS Studio v7.5 (Bioinformatics Solutions, Waterloo, ON, Canada) and searched without taxonomy restriction against Homo Sapiens database (neXtProt Protein reviewed release2019-01). Parent mass error tolerance was set at 10.0 ppm and fragment mass error tolerance was set at 0.5Da. Other searching parameters were trypsin, chymotrypsin and Glu-C specified enzyme for each sample according to the digestion performed and two missed cleavage. Carbamidomethylation of cysteines was set as fixed modification while oxidation of methionines considered as variable modification, three variable PTMs per peptide and one non-specific cleavage. We set the FDR threshold on peptide-spectrum matches (PSM) to 0.1%, resulting in peptide sequence FDR ~0.2%. FDR at protein level was set as <0.2% . All the proteins identified by at least one unique peptide have been filtered out.

The mass spectrometry data have been deposited to the ProteomeXchange Consortium via the PRIDE ([Perez-Riverol et al., 2019](#_ENREF_12)) partner repository with the dataset identifier PXD014201 and PXD014200, for DDA and DIA respectively. Repository dataset raw file legend: OM/OM+IMS: F1 fraction; Mitoplast/IM: F2 fraction; Chymo: Chymotrypsin digestion spectra; Tryp: Trypsin digestion spectra; V8/GluC: GluC digestion spectra; 1: Technical replicate no. 1; 2: Technical replicate no.2; 3: Technical replicate no.3; DDA: Data Dependent Acquisition; DIA: Data Independent Acquisition

**Bioinformatic Data Analysis**

Upon analysis of all spectra, data from F1 and F2 have been split in two groups, and two separate lists of proteins IDs, have been generated. Each list has been obtained by merging datasets of proteins identified in the respective mt-sub fraction with DDA and DIA experiments in sample digested with trypsin, chymotrypsin and GluC proteases. The two protein IDs lists have been used to interrogate IMPI database(Q2 2018) ([Smith and Robinson, 2016](#_ENREF_14)) to obtain the number of entries annotated as mitochondrial.

The identifications of dark proteins were carried out matching our IDs lists with the neXtProt advanced query NXQ_00022 (<http://goo.gl/Wf2Qnn>) which currently contains 1,895 proteins (uPE1, uPE2, uPE3 and uPE4) with no annotated specific function and that account for ~10% of the total number of human proteins, among which 1,254 are classified as uPE1([Paik et al., 2018](#_ENREF_11)). Human Protein Atlas v18.1 ([www.proteinatlas.org](https://v18.proteinatlas.org/)) ([Thul et al., 2017](#_ENREF_15)) was used as reference database for protein localization in Table 1. Grand Average of Hydropathy (GRAVY) scores was calculated according to the Kyte and Doolittle hydropathy scoring system (http://www.gravy-calculator.de/)([Kyte and Doolittle, 1982](#_ENREF_7))

# Supplementary Figures and Tables

## Supplementary Figures

**Supplementary Figure 1.** Venn Diagram of DDA and DIA identifications. **(A)** Venn Diagram between DDA and DIA identification in F1 fraction. **(B)** Venn Diagram between DDA and DIA identification in F2


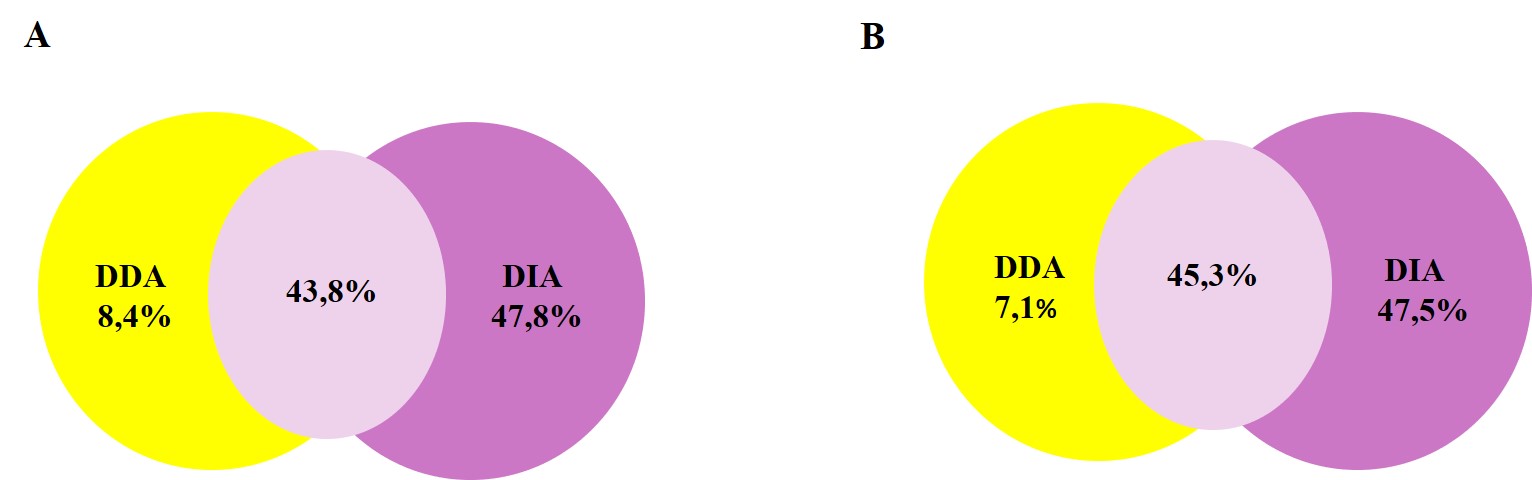


## Supplementary Table Captions

**Tables 1 to 8 are provided separately as file .xlsx**

**Supplementary Table 1:** List of protein identifications provided by Peaks 7.5 for DDA acquisition of trypsin digestion for F1 and F2 fractions.

**Supplementary Table 2:** List of protein identifications provided by Peaks 7.5 for DDA acquisition of Chymotrypsin digestion for F1 and F2 fractions.

**Supplementary Table 3:** List of protein identifications provided by Peaks 7.5 for DDA acquisition of Glu-C digestion for F1 and F2 fractions.

**Supplementary Table 4:** List of protein identifications and quantification provided by PLGS 3.0.3 of DIA acquisition of trypsin digestion for F1 and F2 fractions

**Supplementary Table 5:** List of protein identifications and quantification provided by PLGS 3.0.3 for DIA acquisition of Chymotrypsin digestion for F1 and F2 fractions

**Supplementary Table 6:** List of protein identifications provided by PLGS 3.0.3 for DIA acquisition of Glu-C digestion for F1 and F2 fractions

**Supplementary Table 7:** List of proteins uniquely identified in whole mitochondria (sheet 1), in F1 (sheet 2) and in F2 (sheet 3).

**Supplementary Table 8:** Mitochondrial protein list (Uniprot Accession and Description)

Alberio, T., Pieroni, L., Ronci, M., Banfi, C., Bongarzone, I., Bottoni, P., Brioschi, M., Caterino, M., Chinello, C., Cormio, A., Cozzolino, F., Cunsolo, V., Fontana, S., Garavaglia, B., Giusti, L., Greco, V., Lucacchini, A., Maffioli, E., Magni, F., Monteleone, F., Monti, M., Monti, V., Musicco, C., Petrosillo, G., Porcelli, V., Saletti, R., Scatena, R., Soggiu, A., Tedeschi, G., Zilocchi, M., Roncada, P., Urbani, A., and Fasano, M. (2017). Toward the Standardization of Mitochondrial Proteomics: The Italian Mitochondrial Human Proteome Project Initiative. *J Proteome Res* 16**,** 4319-4329.

Distler, U., Kuharev, J., Navarro, P., Levin, Y., Schild, H., and Tenzer, S. (2014). Drift time-specific collision energies enable deep-coverage data-independent acquisition proteomics. *Nature methods* 11**,** 167.

Distler, U., Kuharev, J., Navarro, P., and Tenzer, S. (2016). Label-free quantification in ion mobility-enhanced data-independent acquisition proteomics. *Nat Protoc* 11**,** 795-812.

Frezza, C., Cipolat, S., and Scorrano, L. (2007). Organelle isolation: functional mitochondria from mouse liver, muscle and cultured fibroblasts. *Nat Protoc* 2**,** 287-295.

Gaudet, P., Michel, P.A., Zahn-Zabal, M., Britan, A., Cusin, I., Domagalski, M., Duek, P.D., Gateau, A., Gleizes, A., Hinard, V., Rech De Laval, V., Lin, J., Nikitin, F., Schaeffer, M., Teixeira, D., Lane, L., and Bairoch, A. (2017). The neXtProt knowledgebase on human proteins: 2017 update. *Nucleic Acids Res* 45**,** D177-D182.

Greco, V., Spalloni, A., Corasolla Carregari, V., Pieroni, L., Persichilli, S., Mercuri, N.B., Urbani, A., and Longone, P. (2018). Proteomics and Toxicity Analysis of Spinal-Cord Primary Cultures upon Hydrogen Sulfide Treatment. *Antioxidants (Basel)* 7.

Kyte, J., and Doolittle, R.F. (1982). A simple method for displaying the hydropathic character of a protein. *J Mol Biol* 157**,** 105-132.

Lane, L., Bairoch, A., Beavis, R.C., Deutsch, E.W., Gaudet, P., Lundberg, E., and Omenn, G.S. (2013). Metrics for the Human Proteome Project 2013–2014 and strategies for finding missing proteins. *Journal of proteome research* 13**,** 15-20.

Martelli, C., Marzano, V., Marini, F., Scotognella, T., Fratoddi, I., Venditti, I., Rotili, D., Solfaroli-Camillocci, E., Collamati, F., Mancini-Terracciano, C., Morganti, S., Maccora, D., Faccini, R., Cartoni, A., Giordano, A., and Castagnola, M. (2018). Mass spectrometry characterization of DOTA-Nimotuzumab conjugate as precursor of an innovative beta(-) tracer suitable in radio-guided surgery. *J Pharm Biomed Anal* 156**,** 8-15.

Nishimura, N., Yano, M. (2014). Separation of the Inner and Outer Mitochondrial Membrane in HeLa Cells *bio-protocol* Vol 4, November 20, 2014.

Paik, Y.K., Overall, C.M., Corrales, F., Deutsch, E.W., Lane, L., and Omenn, G.S. (2018). Toward Completion of the Human Proteome Parts List: Progress Uncovering Proteins That Are Missing or Have Unknown Function and Developing Analytical Methods. *J Proteome Res* 17**,** 4023-4030.

Perez-Riverol, Y., Csordas, A., Bai, J., Bernal-Llinares, M., Hewapathirana, S., Kundu, D.J., Inuganti, A., Griss, J., Mayer, G., Eisenacher, M., Perez, E., Uszkoreit, J., Pfeuffer, J., Sachsenberg, T., Yilmaz, S., Tiwary, S., Cox, J., Audain, E., Walzer, M., Jarnuczak, A.F., Ternent, T., Brazma, A., and Vizcaino, J.A. (2019). The PRIDE database and related tools and resources in 2019: improving support for quantification data. *Nucleic Acids Res* 47**,** D442-D450.

Silva, J.C., Gorenstein, M.V., Li, G.Z., Vissers, J.P., and Geromanos, S.J. (2006). Absolute quantification of proteins by LCMSE: a virtue of parallel MS acquisition. *Mol Cell Proteomics* 5**,** 144-156.

Smith, A.C., and Robinson, A.J. (2016). MitoMiner v3.1, an update on the mitochondrial proteomics database. *Nucleic Acids Res* 44**,** D1258-1261.

Thul, P.J., Akesson, L., Wiking, M., Mahdessian, D., Geladaki, A., Ait Blal, H., Alm, T., Asplund, A., Bjork, L., Breckels, L.M., Backstrom, A., Danielsson, F., Fagerberg, L., Fall, J., Gatto, L., Gnann, C., Hober, S., Hjelmare, M., Johansson, F., Lee, S., Lindskog, C., Mulder, J., Mulvey, C.M., Nilsson, P., Oksvold, P., Rockberg, J., Schutten, R., Schwenk, J.M., Sivertsson, A., Sjostedt, E., Skogs, M., Stadler, C., Sullivan, D.P., Tegel, H., Winsnes, C., Zhang, C., Zwahlen, M., Mardinoglu, A., Ponten, F., Von Feilitzen, K., Lilley, K.S., Uhlen, M., and Lundberg, E. (2017). A subcellular map of the human proteome. *Science* 356.

Vissers, J.P., Langridge, J.I., and Aerts, J.M. (2007). Analysis and quantification of diagnostic serum markers and protein signatures for Gaucher disease. *Mol Cell Proteomics* 6**,** 755-766.
